# p39-associated Cdk5 activity regulates dendritic morphogenesis

**DOI:** 10.1038/s41598-020-75264-6

**Published:** 2020-10-30

**Authors:** Li Ouyang, Yu Chen, Ye Wang, Yuewen Chen, Amy K. Y. Fu, Wing-Yu Fu, Nancy Y. Ip

**Affiliations:** 1 Division of Life Science, State Key Laboratory of Molecular Neuroscience and Molecular Neuroscience Center, The Hong Kong University of Science and Technology, Clear Water Bay, Hong Kong, China; 2 Hong Kong Center for Neurodegenerative Diseases, Hong Kong Science Park, Hong Kong, China; 3 The Brain Cognition and Brain Disease Institute, Shenzhen Institute of Advanced Technology, Chinese Academy of Sciences, Shenzhen-Hong Kong Institute of Brain Science-Shenzhen Fundamental Research Institutions, Shenzhen, 518055 Guangdong China; 4 Guangdong Provincial Key Laboratory of Brain Science, Disease and Drug Development, HKUST Shenzhen Research Institute, Shenzhen–Hong Kong Institute of Brain Science, Shenzhen, 518057 Guangdong China

**Keywords:** Cellular neuroscience, Development of the nervous system

## Abstract

Dendrites, branched structures extending from neuronal cell soma, are specialized for processing information from other neurons. The morphogenesis of dendritic structures is spatiotemporally regulated by well-orchestrated signaling cascades. Dysregulation of these processes impacts the wiring of neuronal circuit and efficacy of neurotransmission, which contribute to the pathogeneses of neurological disorders. While Cdk5 (cyclin-dependent kinase 5) plays a critical role in neuronal dendritic development, its underlying molecular control is not fully understood. In this study, we show that p39, one of the two neuronal Cdk5 activators, is a key regulator of dendritic morphogenesis. Pyramidal neurons deficient in p39 exhibit aberrant dendritic morphology characterized by shorter length and reduced arborization, which is comparable to dendrites in Cdk5-deficient neurons. RNA sequencing analysis shows that the adaptor protein, WDFY1 (WD repeat and FYVE domain-containing 1), acts downstream of Cdk5/p39 to regulate dendritic morphogenesis. While WDFY1 is elevated in p39-deficient neurons, suppressing its expression rescues the impaired dendritic arborization. Further phosphoproteomic analysis suggests that Cdk5/p39 mediates dendritic morphogenesis by modulating various downstream signaling pathways, including PI3K/Akt-, cAMP-, or small GTPase-mediated signaling transduction pathways, thereby regulating cytoskeletal organization, protein synthesis, and protein trafficking.

## Introduction

Neurons are polarized cells with a single axon, which transmits signals, and multiple dendrites, which receive and process information from presynaptic axonal inputs. Each subtype of neurons has unique dendritic morphology, which ensures that the neuronal circuits are appropriately wired with a spatiotemporally optimal number of synaptic connections and a proper receptive field for appropriate neuronal firing^1^. Dendritic morphology is tightly controlled by well-orchestrated signaling events both during development and in the mature brain^2,3^. These external stimuli-mediated signaling pathways instruct the organization of dendritic trees by regulating gene transcription and protein synthesis, controlling protein and lipid trafficking along the dendrites, and coordinating microtubule- or actin-binding proteins to regulate cytoskeletal assembly and disassembly^3^. Accordingly, protein kinases play central roles in coordinating the activities of these cellular processes.

Cdk5 (cyclin-dependent kinase 5) is a proline-directed serine/threonine kinase that is ubiquitously expressed in the central nervous system^4,5^. Cdk5 possesses kinase activity that requires the binding of one of its two specific activators, p39 or p35^6–8^. Cdk5 plays a pivotal role during neural development by participating in neuronal differentiation, neuronal migration, axonal guidance, and dendritic morphogenesis as well as various aspects of neuronal functions such as synaptic plasticity and neurotransmission^9,10^. Suppression of Cdk5 expression in neurons results in simplified dendritic trees^11^, whereas inhibition of Cdk5 by S-nitrosylation promotes dendritic complexity^12^. Furthermore, the neuronal activity-stimulated nuclear translocation of Cdk5 regulates the expression of genes for dendrite outgrowth^13^. Thus, dendritic development requires the precise control of Cdk5 activity. Accordingly, various proteins involved in the regulation of dendritic development and maintenance, including TrkB (tropomyosin receptor kinase B), microtubule-associated proteins (MAPs), and CRMP2 (collapsin response mediator protein-2), are also Cdk5 substrates^14–17^.

The Cdk5 activators, p35 and p39, share approximately 60% sequence homology and exhibit differential developmental expression in the brain^8^. The expression of p35 protein is high throughout the embryonic stage, whereas that of p39 increases during postnatal differentiation^4^. Mice lacking both p35 and p39 exhibit inverted cortical lamination, aberrant neuronal morphology, and defective synaptic functions, which are also observed in Cdk5-knockout mice^18,19^. These observations suggest that the activity of Cdk5 is attributable to its association with these two activators, which might have overlapping functions. However, knockout of p35 but not p39 results in inverted cortical lamination^20^. Meanwhile, p39 knockout results in impaired axonal branching and dendritic spine morphogenesis^21^. Although in vitro experiments suggest that p35 and p39 share similar substrate specificity, they are spatially segregated within neurons and have different biochemical properties^22,23^. Compared to p35, p39 exhibits slower degradation, higher membrane binding and nuclear accumulation, and lower affinity for Cdk5^22–24^. Therefore, Cdk5/p35 and Cdk5/p39 complexes likely have distinct functional roles by targeting specific substrates in the nervous system. However, as most previous studies have focused on Cdk5/p35, the roles of Cdk5/p39 in brain development and functioning are less understood^9,10^.

In this study, we show that p39-associated Cdk5 activity is required for dendritic development. Depletion of p39 but not p35 phenocopies Cdk5-deficient neurons, which results in shorter and fewer dendrites compared to wild-type neurons. Moreover, RNA sequencing and mass spectrometry analyses of phosphoproteins suggest that p39 regulates dendritic complexity by modulating the expression of a signaling adaptor protein, WDFY1 (WD repeat and FYVE domain-containing protein 1). Specifically, suppression of WDFY1 rescues the defective dendritic morphology in p39-deficient neurons. Furthermore, we identify multiple phosphorylation-dependent signaling pathways that regulate cytoskeletal dynamics as well as protein synthesis and trafficking in p39-dependent dendritic morphogenesis.

## Results

### Cdk5/p39 activity is required for dendritic development

**Figure 1:**
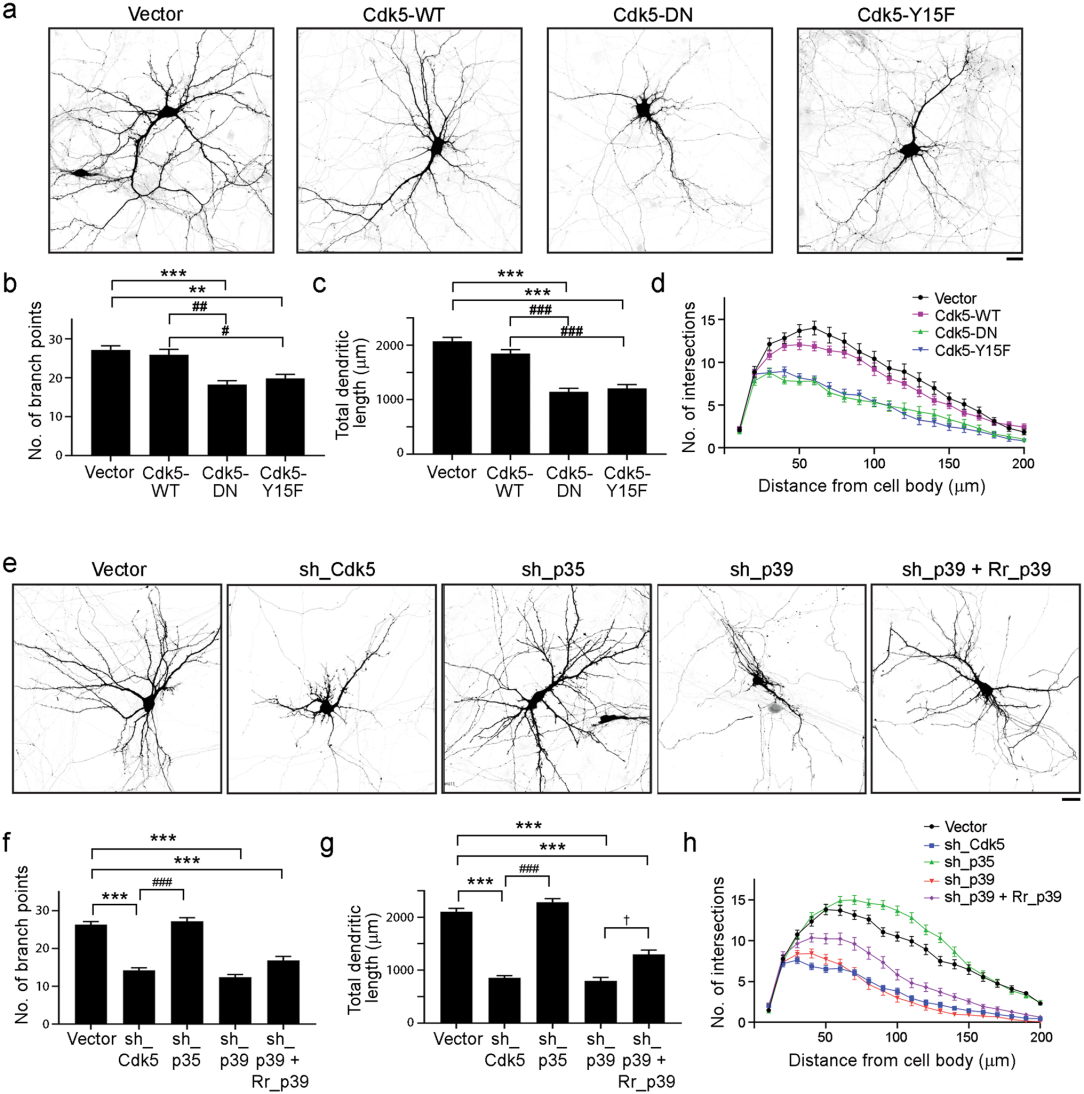
p39-associated Cdk5 activity is important for dendritic development. (**a**–**d**) Cdk5 activity is required for proper dendritic development. Rat hippocampal neurons at 7 days in vitro (DIV) were transfected with vector control or the indicated Cdk5 cDNA constructs, and dendritic morphology was examined at 14 DIV. (**a**) Representative images of wild-type neurons (Cdk5-WT) and Cdk5 activity-deficient neurons (Cdk5-DN or Cdk5-Y15F). Dendritic complexity was analyzed by quantifying total dendrite number (**b**) and total dendrite length (**c**), and Sholl analysis (**d**). Scale bar: 20 μm; *n* = 17–46 neurons from 3 independent experiments; **p < 0.01, ***p < 0.001 versus Mock; ^#^*p* < 0.05, ^##^*p* < 0.01, ^###^*p* < 0.001 versus Cdk5-WT; Kruskal–Wallis one-way ANOVA followed by Dunn’s test. (**e**–**h**) Reduced dendritic complexity in Cdk5-, p35-, or p39-knockdown neurons. Rat hippocampal neurons at 7 DIV were transfected with vector control or shRNA targeting Cdk5, p35, p39, or p39 together with RNAi-resistant p39 mutant (Rr_p39). Dendritic morphology was examined at 14 DIV. Representative images (**e**), quantification of total dendrite number (**f**) and total dendrite length (**g**), and Sholl analysis (**h**). Scale bar: 20 µm; *n* = 30–35 neurons from 3 independent experiments; ***p < 0.001 versus Vector; ^###^*p* < 0.001 versus sh_Cdk5; ^†^*p* < 0.05 versus sh_p39; Kruskal–Wallis one-way ANOVA followed by Dunn’s test.

As a first step to investigate how Cdk5 regulates dendritic morphogenesis, we overexpressed wild-type Cdk5 (Cdk5-WT) or dominant-negative Cdk5 (Cdk5-DN) in cultured rat hippocampal neurons at 7 days in vitro (DIV) and examined dendritic phenotypes at 14 DIV. Neurons expressing Cdk5-DN exhibited defective dendritic morphology as indicated by reductions of the total number of dendrites, dendrite length, and dendritic complexity (Fig. 1a–d). Various extracellular stimuli phosphorylate the Y15 residue of Cdk5, which augments Cdk5 kinase activity^25,26^. Similar to neurons expressing Cdk5-DN, neurons expressing Cdk5-Y15F mutant exhibited shorter and fewer dendrites compared to those expressing Cdk5-WT (Fig. 1a–d).

**Figure 2:**
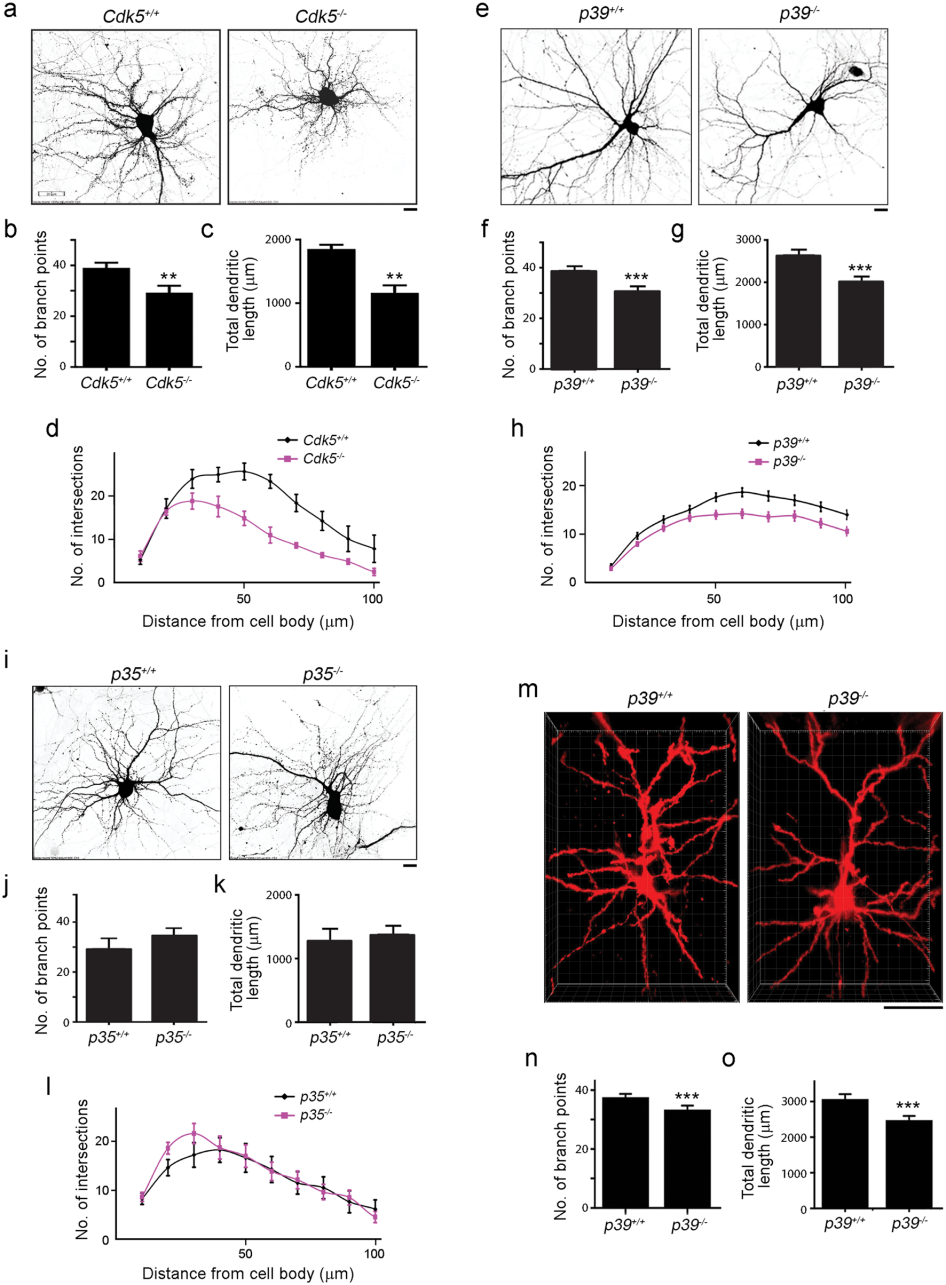
Impaired dendritic morphogenesis in *Cdk5*^−/−^, *p39*^−/−^, and *p35*^−/−^ neurons. (**a**–**d**) Dendritic morphology of *Cdk5*^+/+^ and *Cdk5*^−/−^ hippocampal neurons at 14 days in vitro (DIV). Representative images (**a**) and quantification of total dendrite number (**b**) and total dendrite length (**c**), and Sholl analysis (**d**) of *Cdk5*^+/+^ and *Cdk5*^−/−^ neurons. Scale bar: 10 µm; *n* = 7–8 neurons; **p < 0.01, Mann–Whitney *U-*test. (**e**–**h**) Dendritic morphology of *p39*^+/+^ and *p39*^−/−^ hippocampal neurons at 14 DIV. Representative images (**e**) and quantification of dendrite number (**f**) and total dendrite length (**g**), and Sholl analysis (**h**) of *p39*^+/+^ and *p39*^−/−^ neurons. Scale bar: 10 µm; *n* = 30 neurons; ***p < 0.001, Mann–Whitney *U-*test. (**i**–**l**) Dendritic morphology of *p35*^+/+^ and *p35*^−/−^ hippocampal neurons at 14 DIV. Representative images (**i**) and quantification of dendrite number (**j**) and total dendrite length (**k**), and Sholl analysis (**l**) of *p35*^+/+^ and *p35*^−/−^ neurons. Scale bar: 10 µm; *n* = 7–8 neurons. (**m**–**o**) Dendritic morphology of pyramidal neurons in the cerebral cortex of 1-month-old *p39*^+/+^ and *p39*^−/−^ mice. Representative images (**m**) and quantification of dendrite number (**n**) and total dendrite length (**o**). Scale bar: 50 µm; *n* = 22–29 neurons from 3 mice; ***p < 0.001, Mann–Whitney *U-*test.

Next, we determined whether p35- or p39-associated Cdk5 activity regulates dendritic morphogenesis. Knockdown of endogenous p39 in cultured hippocampal pyramidal neurons reduced dendritic complexity (i.e., shorter and simpler dendritic branching), which was comparable to that in Cdk5-deficient neurons (Fig. 1e–h). Re-expression of the RNAi-resistant form of p39 in p39-knockdown neurons rescued this defective dendritic phenotype, resulting in longer total dendrite length and more complex dendritic trees (Fig. 1e–h). In contrast, p35-knockdown hippocampal neurons exhibited slightly enhanced dendritic complexity (Fig. 1e–h) compared to control neurons, suggesting that either p35 is dispensable for dendritic development or that p39 alone can sufficiently compensate for the loss of p35. Similarly, cultured hippocampal pyramidal neurons derived from *p39*^−/−^ mice also exhibited deficits in the development of dendritic trees, although the impairment was not as severe as that observed in *Cdk5*^−/−^ mice (Fig. 2a–h); meanwhile, dendritic trees appeared to be normal in cultured hippocampal pyramidal neurons derived from *p35*^−/−^ mice (Fig. 2i–l). Of note, we observed more severe dendritic defects in p39-knockdown neurons than *p39*^−/−^ neurons, which might be due to the activities of compensatory signaling pathways as a result of germline knockout of p39.

To examine the role of p39 in dendritic development in vivo, we examined the dendritic morphology of pyramidal neurons in *p39*^−/−^ mouse brains. Three-dimensional reconstruction of the dendritic trees of Golgi-stained *p39*^−/−^ cortical pyramidal neurons showed that these neurons also exhibited reduced dendritic complexity characterized by shorter and fewer dendrites (Fig. 2m–o). Taken together, these results suggest that the Cdk5 activity associated with p39 but not p35 is the key regulator of dendritic morphogenesis.

### Dendritic development is regulated by p39 through the modulation of WDFY1 expression

**Figure 3:**
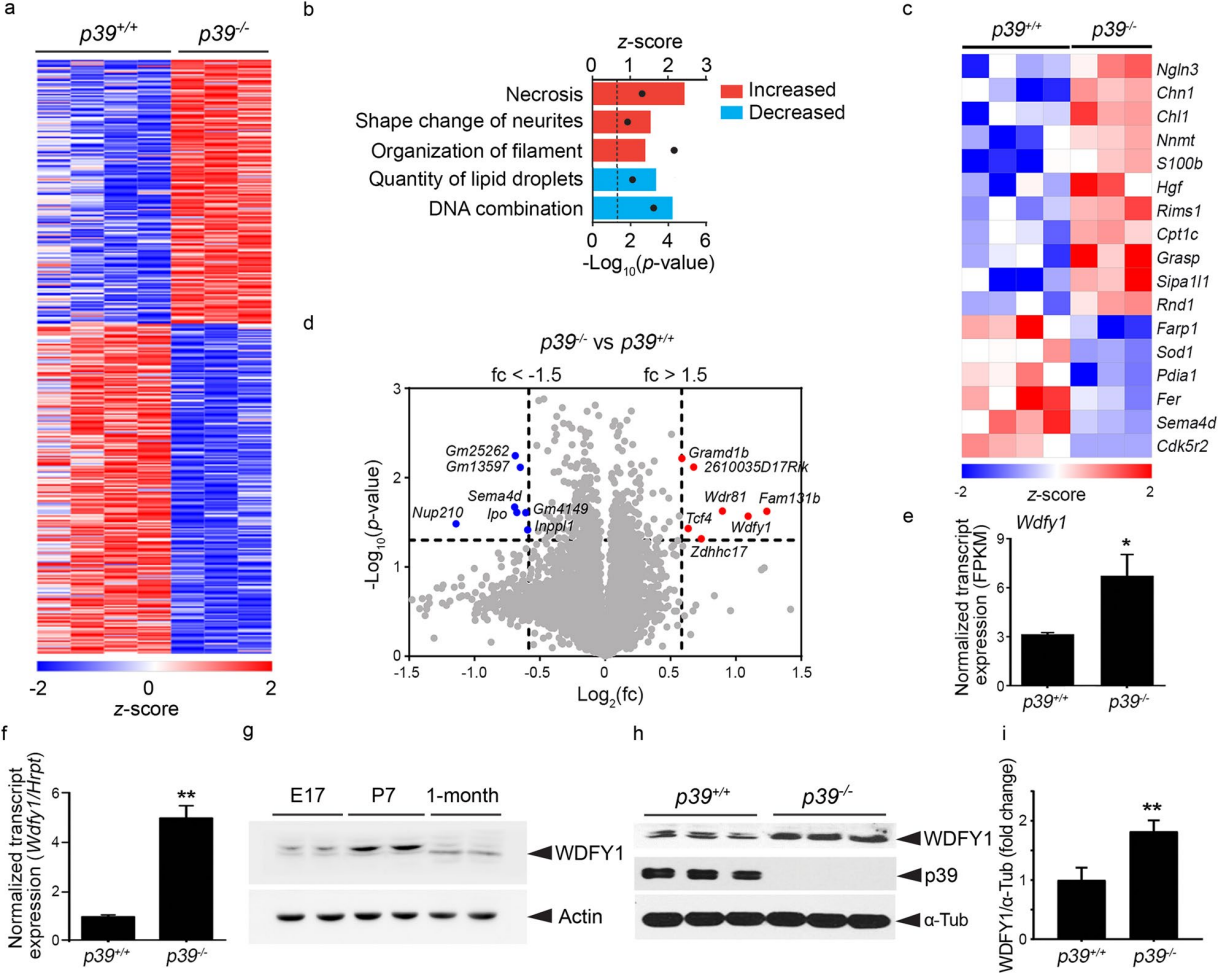
Transcriptome analysis of *p39*^+/+^ and *p39*^−/−^ cortical neurons. (**a**) Heatmap showing the relative expression of differentially expressed genes between *p39*^−/−^ and *p39*^+/+^ cortical neurons. There were 278 upregulated and 361 downregulated genes. The total RNA from *p39*^+/+^ and *p39*^−/−^ cortical neurons was extracted at 10 days in vitro (DIV) for whole-transcriptome analysis. (**b**) Ingenuity pathway analysis (IPA) of the biofunctions of differentially expressed genes between *p39*^−/−^ and *p39*^+/+^ cortical neurons. The activation of biofunctions according to differential gene expression in *p39*^−/−^ and *p39*^+/+^ cortical neurons was determined by the *z*-score algorithm with a criterion of *p* < 0.05 (i.e., − log_10_ ≥ 1.3; black dots) using Fisher’s exact test. (**c**) Heatmap showing the differential gene expression of the “shape change of the neurite” group between *p39*^+/+^ and *p39*^−/−^ cortical neurons. (**d**) Volcano plot showing the log_2_ fold change and − log_10_(*p *value) of each gene comparing *p39*^−/−^ and *p39*^+/+^ cortical neurons. Differentially expressed genes with fold change > 1.5 and <  −1.5 are highlighted in red and blue, respectively. The dashed line indicates *p* < 0.05 and a fold change > 1.5 or <  −1.5. (**e**) *Wdfy1* transcript levels in *p39*^+/+^ and *p39*^−/−^ neurons determined by RNA sequencing analysis. (**f**) Real-time PCR analysis of *Wdfy1* transcript levels in *p39*^+/+^ and *p39*^−/−^ neurons. (**g**) WDFY1 protein expression at different developmental stages—embryonic day (E) 17, E17; postnatal day (P) 7, P7, and 1 month old—in C57/BL6 mouse brains. (**h**, **i**) Elevated WDFY1 protein expression in *p39*^−/−^ mouse brains. Western blot analysis (**h**) and quantification (**i**) of WDFY1 protein in p39-knockout mouse forebrains at P7; *n* = 3 brains; *p < 0.05, **p < 0.01, unpaired Student’s *t*-test.

To examine the molecular mechanism by which p39-associated Cdk5 activity regulates dendritic development, we profiled the transcriptomes of cortical neurons derived from *p39*^−/−^ or *p39*^+/+^ mouse embryos. Compared to cortical neurons from littermate controls, there were 278 upregulated and 361 downregulated genes in *p39*^−/−^ cortical neurons (*p* < 0.05; Fig. 3a). To identify the most differentially regulated cellular processes, we performed ingenuity pathway analysis (IPA) of biofunctions including “molecular and cellular functions” and “physiological system development and function.” In IPA, the most regulated processes were determined on the basis of the overlap of the activation *z*-score algorithm (with a 1.2-fold change cutoff) and *p* < 0.05. We found that the most prominent cellular processes regulated in p39-deficient neurons involved (1) the “shape change of neurites,” (2) “organization of filaments,” and (3) “quantity of lipid droplets” (Fig. 3b). These results suggest that p39 might regulate neuronal morphogenesis by modulating the cellular processes that control the assembly and organization of subcellular components. Specifically, some of the differentially regulated genes associated with the cellular process “shape change of neurites,” including *Farp1* (Ferm, ARHGEF and pleckstrin domain-containing protein 1; a Rac1 activator), *Rnd1* (Rnd1; a Rho GTPase), *Chn1* (α-chimaerin; a Rho GTPase-activating protein), *Sema4D* (Semaphorin 4D), and *hgf* (hepatocyte growth factor), are involved in the regulation of dendritic complexity (Fig. 3c)^27–31^.

To identify specific genes involved in Cdk5/p39-regulated dendritic development, we generated a volcano plot to show the most differentially expressed genes (i.e., genes with a 1.5-fold change cutoff and *p* < 0.05) between *p39*^−/−^ and wild-type neurons (Fig. 3d). Among them, the transcript level of *Wdfy1* was 1.7 fold higher in *p39*^−/−^ neurons (Fig. 3e); of note, *Wdfy1* is an adaptor protein in certain signaling pathways and is reported to regulate the differentiation of the neural cell lineage^32–34^. Real-time PCR confirmed the increase of *Wdfy1* transcript in *p39*^−/−^ neurons (4.7-fold increase; Fig. 3f). WDFY1 protein expression was elevated in the mouse forebrain at postnatal stages and decreased in the adult cerebral cortex (Fig. 3g). Compared to that in the wild-type control, WDFY1 protein level was 1.8 fold higher in the forebrain in *p39*^−/−^ mice at postnatal day 7 (Fig. 3h, i), which is the critical period for dendritic development; similar regulation was not observed in the embryonic forebrain (Supplementary Fig. S1a,S1b) or the cerebral cortex at 1 month (Supplementary Fig. S1c,S1d) in *p39*^−/−^ mice. These results suggest that p39 regulates WDFY1 protein expression in the brain during the period critical for dendritic development.

**Figure 4:**
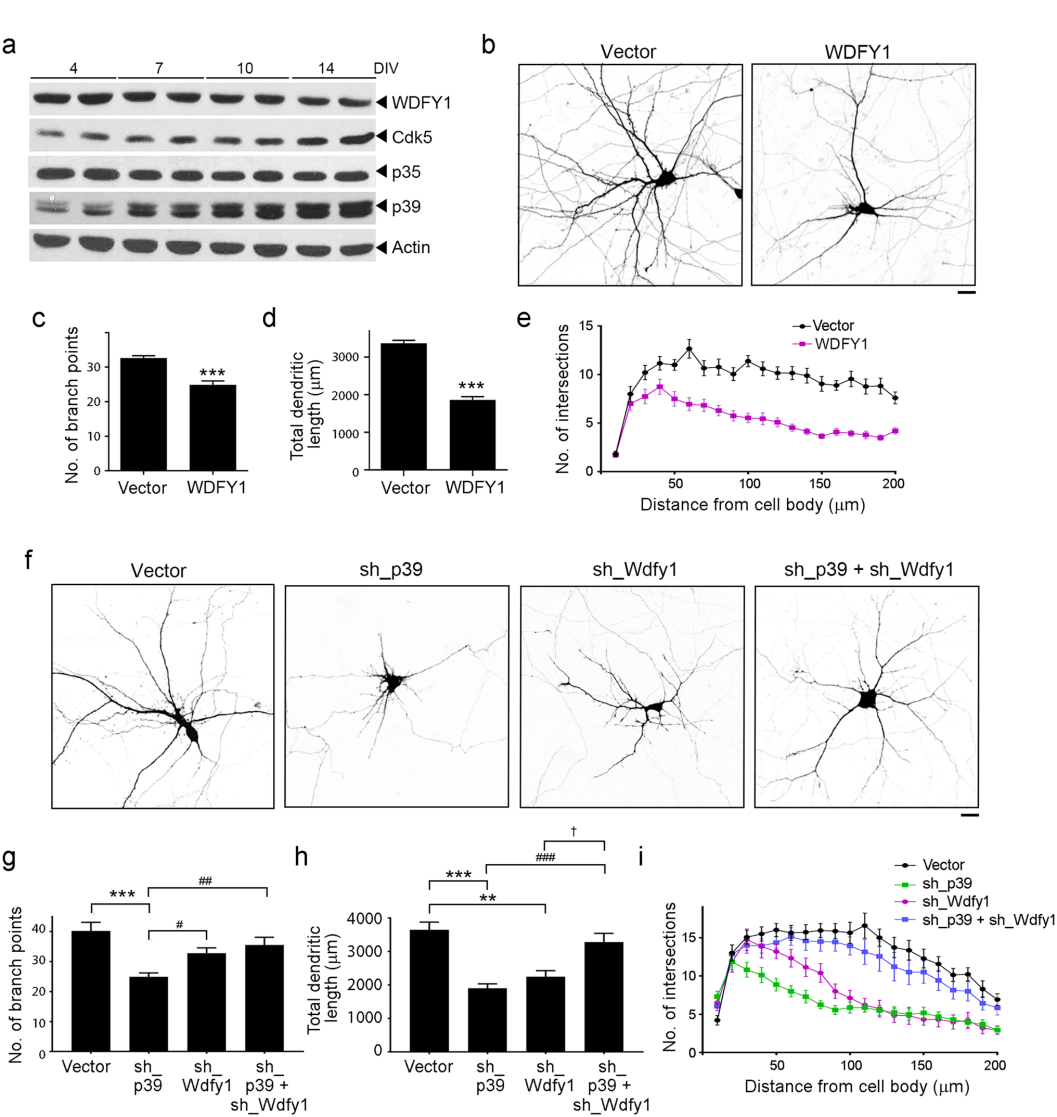
Suppression of WDFY1 expression restores impaired dendritic development in p39-deficient neurons. (**a**) Developmental expression of WDFY1 in rat cortical neurons at 4, 7, 10 and 14 days in vitro (DIV). (**b**–**e**) Elevated WDFY1 protein expression impaired dendritic development in hippocampal neurons. Rat hippocampal neurons at 7 DIV were transfected with vector control or *Wdfy1* cDNA construct, and dendritic morphology was examined at 14 DIV. (**b**) Representative images showing the dendritic morphology of WDFY1-overexpressing hippocampal neurons. (**c**–**e**) Quantification of dendrite number (c) and total dendrite length (**d**), and Sholl analysis (**e**). Scale bar: 20 µm; *n* = 18–20 neurons from 3 independent experiments; ***p < 0.001, Mann–Whitney *U*-test. (**f**–**i**) Suppression of *Wdfy1* restored the dendritic morphology in *p39*-deficient hippocampal neurons. Rat hippocampal neurons at 7 DIV were transfected with pSUPER vector control and shRNA targeting *p39*, *Wdfy1*, or both, and dendritic morphology was examined at 14 DIV. (**f**) Representative images showing dendritic morphology. (**g**–**i**) Quantification of the number of dendrites (**g**) and total dendrite length (h), and Sholl analysis (i); *n* = 14–16 neurons from 3 independent experiments; **p < 0.01, ***p < 0.001 versus Vector; ^#^*p* < 0.05, ^##^*p* < 0.01, ^###^*p* < 0.001 versus sh_p39; ^†^*p* < 0.05 versus sh_Wdfy1; Kruskal–Wallis one-way ANOVA followed by Dunn’s test.

We then determined whether WDFY1 protein is involved in dendritic development in cultured rat neurons. WDFY1 protein was stably expressed in cultured cortical neurons throughout development (Fig. 4a). Meanwhile, overexpression of WDFY1 protein in hippocampal neurons impaired dendritic development (Fig. 4b–e). However, the dendritic trees of WDFY1-deficient hippocampal neurons exhibited defective dendritic morphology with respect to total dendrite length but not dendritic branching (Fig. 4f–i). These results suggest that the maintenance of an optimal level of WDFY1 is critical for dendritic morphogenesis.

We subsequently examined whether suppressing the enhanced WDFY1 expression in p39-deficient hippocampal neurons rescues the defective dendritic development. Interestingly, compared to p39-knockdown and WDFY1-knockdown neurons, which both exhibited reduced total dendritic length and dendritic complexity, these defective dendritic phenotypes were rescued in neurons lacking both WDFY1 and p39 (Fig. 4f–i). Moreover, the total dendrite length and dendritic complexity in p39/WDFY1-deficient neurons were comparable to those in control neurons, although the dendrites were thinner in the former (Fig. 4f–i). Thus, our results collectively indicate that WDFY1 at least in part mediates the downstream signaling of p39 in dendritic development.

### Identification of Cdk5/p39 protein substrates and downstream signaling processes

**Figure 5:**
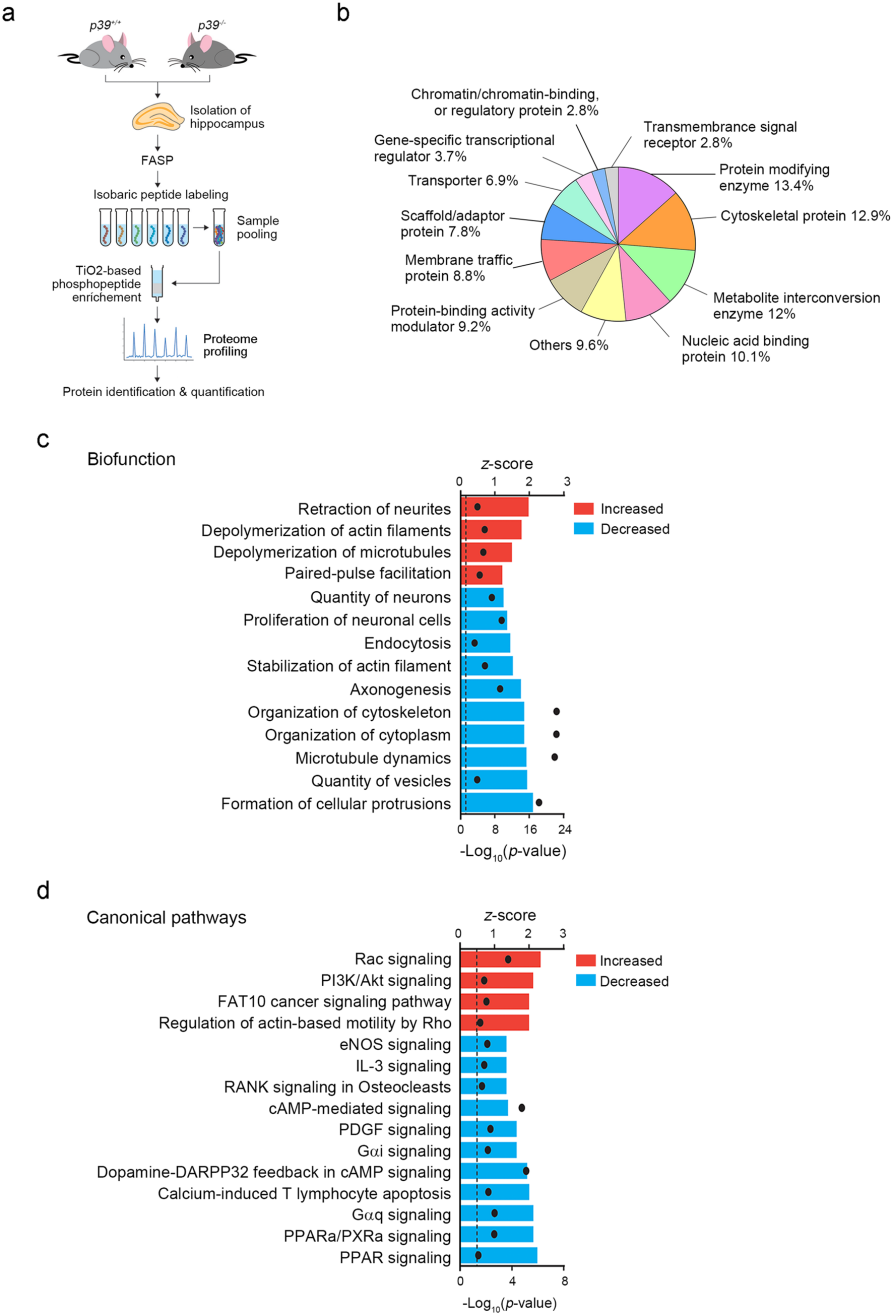
Phosphoproteomic analysis of the hippocampi of 1-month-old *p39*^+/+^ and *p39*^−/−^ mice. (**a**) Workflow of phosphoproteomic analyses between *p39*^+/+^ and *p39*^−/−^ hippocampi. (**b**) Gene Ontology (GO) analysis according to the PANTHER classification system revealed the protein classification of differentially phosphorylated proteins between *p39*^+/+^ and *p39*^−/−^ mouse hippocampi. (**c**) Ingenuity pathway analysis (IPA) of the biofunctions of differentially regulated phosphoproteins in *p39*^+/+^ and *p39*^−/−^ mouse hippocampi. The activation of biofunctions based on the differentially regulated phosphoproteins between *p39*^+/+^ and *p39*^−/−^ hippocampi was determined by the *z*-score algorithm with a criterion of *p* < 0.05 (i.e., − log_10_ ≥ 1.3; black dots) using Fisher’s exact test. (**d**) IPA of the canonical pathways of differentially regulated phosphoproteins between *p39*^+/+^ and *p39*^−/−^ mouse hippocampi. The activation of canonical pathways based on the differentially phosphorylated protein expression between *p39*^+/+^ and *p39*^−/−^ hippocampi was determined by the *z*-score algorithm with a criterion of *p* < 0.05 (i.e., − log_10_ ≥ 1.3; black dots) using Fisher’s exact test.

Neuronal morphogenesis involves the coordination and regulation of multiple intracellular signaling pathway components including intracellular kinases, second messengers, small GTPases, and cytoskeletal regulatory proteins^35,36^. The activities of these intracellular signaling components are largely dependent on their phosphorylation status. Therefore, to examine the molecular basis by which p39 regulates dendritic morphogenesis, we investigated the changes in the phosphorylated protein profile of p39-knockout mouse brains. Specifically, we compared the differentially phosphorylated peptides (i.e., phosphopeptides) in the hippocampi between *p39*^−/−^ mice and their wild-type littermates by using isobaric tags for relative and absolute quantification (iTRAQ)-based proteomics analysis (Fig. 5a). Accordingly, we detected 5255 peptides and identified 1188 nonredundant proteins in *p39*^+/+^ and *p39*^−/−^ hippocampi. The 369 downregulated and 241 upregulated phosphopeptides that met the criterion of *p* < 0.05 corresponded to 267 and 194 proteins, respectively. Gene Ontology (GO) analysis based on the PANTHER classification system^37^ showed that the differentially regulated phosphorylated proteins in the *p39*^−/−^ mouse hippocampus were predominantly protein-modifying enzymes followed by cytoskeletal proteins, metabolite interconversion enzymes, and nucleic acid-binding proteins (Fig. 5b).

Among them, 96 downregulated and 69 upregulated phosphopeptides met the criterion of fold change > 1.2 (Tables 1, 2, 3), corresponding to 89 and 66 proteins, respectively. The downregulated phosphorylated proteins, including MAPs, GPRIN1 (G protein-regulated inducer of neurite outgrowth 1), PALM (paralemmin, which is implicated in plasma membrane dynamics), DPYSL2 (CRMP2), and DPYSL3 (CRMP4), are involved in the regulation of the cytoskeletal dynamics that mediate axonal and dendritic morphogenesis^38–41^. Furthermore, 39 of the downregulated phosphorylated proteins contained a predicted phosphopeptide that matched Cdk5 consensus phosphorylation sequences (i.e., serine/threonine-proline [S/TP]), suggesting that they are direct substrates of Cdk5/p39 (Table 1). Nonetheless, only 6 of them—MAP1B, MAP2, CRMP2, CRMP4, GPRIN1, and CaMKV (CaM kinase-like vesicle-associated)—have been reported as Cdk5 substrates (Table 1)^36,42,43^. Interestingly, except for the phosphorylation site of CRMP2, which has been reported to be downregulated in *Cdk5*^−/−^ brains^42^, the predicted phosphorylation sites in 5 of these proteins are novel Cdk5 phosphorylation sites. Of note, the phosphorylation of WDFY1 at its consensus Cdk5 phosphorylation sequence, proline-directed Ser408, was significantly upregulated in p39-knockout mouse hippocampi (Table 3) as indicated by mass spectrometry analysis. These findings suggest that WDFY1 might be phosphorylated by other proline-directed serine/threonine kinases in p39-knockout mouse brains. Taken together, our results suggest that Cdk5/p39 activity not only impacts *Wdfy1* transcript level, but also affects the phosphorylation status of WDFY1 protein.

**Table 1:**
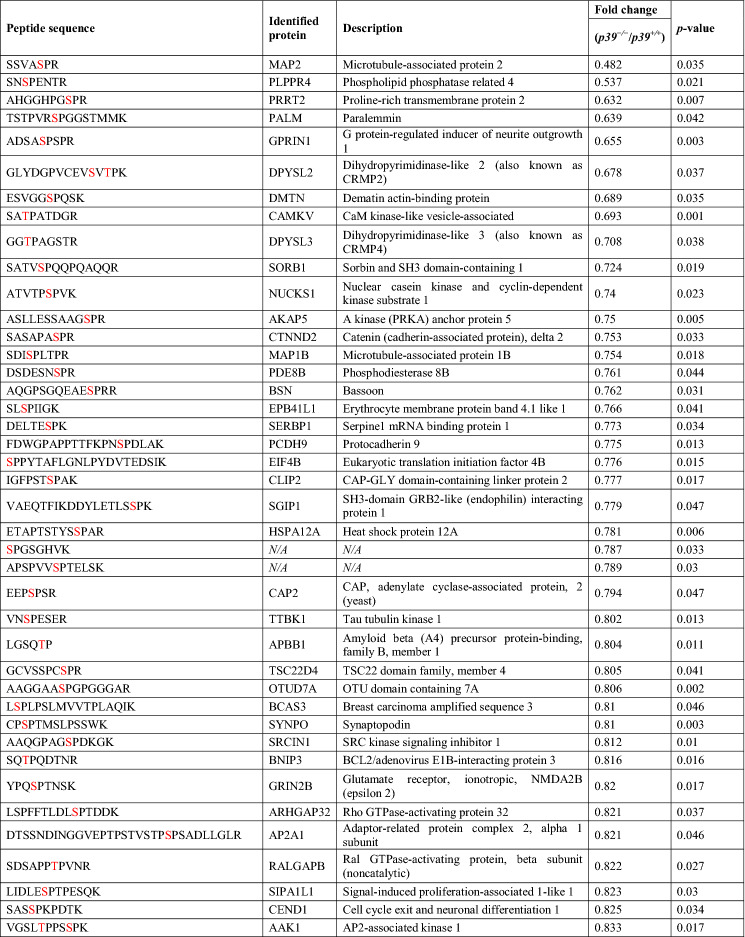
Downregulated phosphorylated proteins that contain serine/threonine-proline motifs in *p39*^−/−^ mice.

The predicted phosphorylation residues are indicated in red (*p* < 0.05).

**Table 2:**
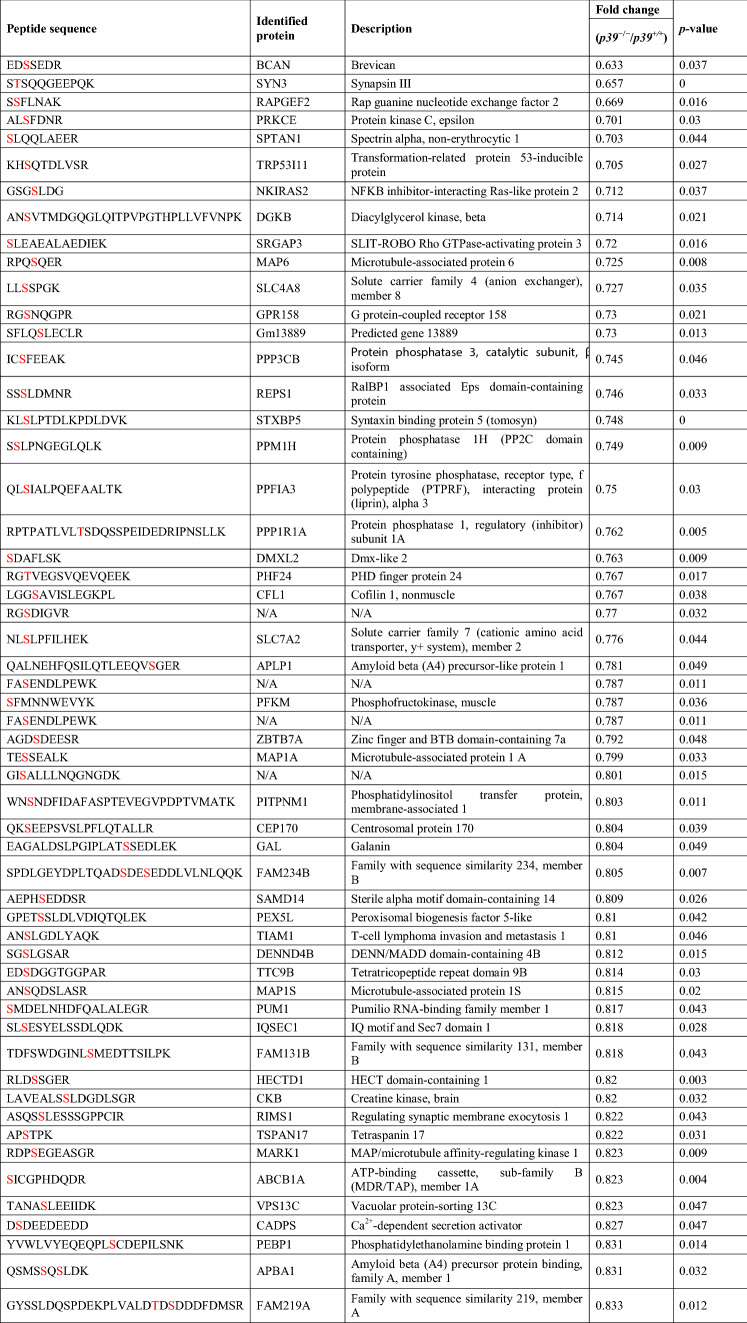
Downregulated phosphorylated proteins that do *not* contain serine/threonine-proline motifs in *p39*^−/−^ mice.

The predicted phosphorylation residues are indicated in red (*p* < 0.05).

**Table 3:**
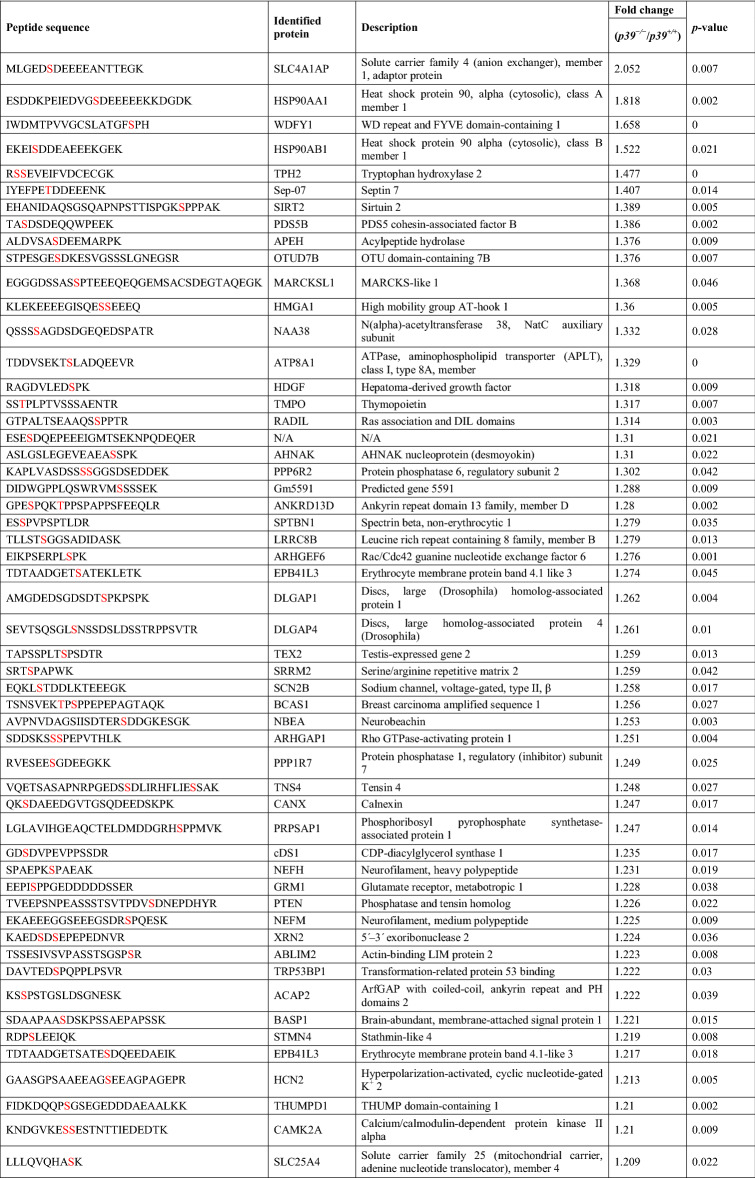

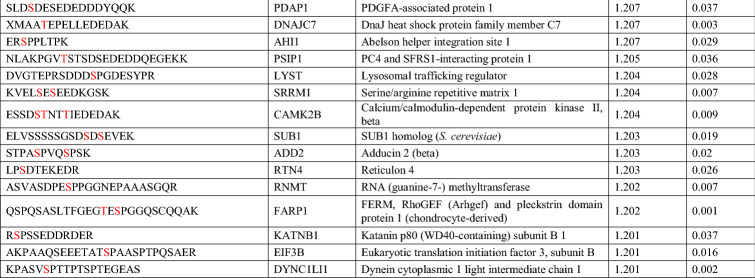
Upregulated phosphorylated proteins in *p39*^−/−^ mice.

The predicted phosphorylation residues are indicated in red (*p* < 0.05).

To further examine the molecular pathways regulated by p39 in neurons, we submitted the differentially regulated phosphoproteins to IPA of biofunctions and canonical pathways. The most regulated processes were determined on the basis of the overlap of the activation *z*-score algorithm (with a 1.5-fold change cutoff) and *p* < 0.05. Concordant with the effects of p39 on dendritic morphology, IPA of biofunctions revealed that in *p39*^−/−^ hippocampi, the most upregulated cellular processes were (1) “retraction of neurites,” (2) “depolymerization of actin filaments,” and (3) “depolymerization of microtubules.” Meanwhile, the most downregulated cellular processes were (1) “formation of cellular protrusions,” (2) “quantity of vesicles,” (3) “microtubule dynamics,” (4) “organization of cytoplasm,” (5) “organization of cytoskeleton,” and (6) “axonogenesis” (Fig. 5c). Thus, these findings suggest that Cdk5/p39 regulates the growth and branching of dendrites through the regulation of cytoskeletal organization and stabilization as well as protein synthesis and trafficking. Furthermore, IPA of canonical pathways revealed the most upregulated signaling pathways in *p39*^−/−^ hippocampi were (1) “Rac signaling,” (2) “PI3K/Akt signaling,” (3) “FAT10 cancer signaling pathway,” and (4) “regulation of actin-based motility by Rho” (Fig. 5d), while the most downregulated signaling pathways were (1) “PPAR-related signalings,” (2) “heterotrimeric G proteins” (i.e., G_α/q_ & G_αi_), and (3) “cAMP-related pathways” (Fig. 5d).

## Discussion

Cdk5 is a pleiotropic kinase that regulates multiple cellular processes by specifically associating with one of its two activators, p35 or p39. While the role of Cdk5/p35 in brain development is well characterized, the functional roles and molecular basis of Cdk5/p39 remain largely unclear^9,10^. Accordingly, in this study, we demonstrate that p39 but not p35 is indispensable for dendritic development, thus revealing a nonoverlapping function of the two Cdk5 activators in brain development. Moreover, our transcriptomic and phosphoproteomic analyses suggest that Cdk5/p39 regulates dendritic development through the modulation of multiple intracellular signaling complexes that regulate the organization and stability of the actin and microtubule network.

While Cdk5 is a critical kinase that regulates neuronal functions, most studies have focused on the regulation and roles of p35-associated Cdk5 activity^9,10^. However, as p39 is the major Cdk5 activator expressed in the brain after postnatal development^4^, Cdk5 activity in postnatal and adult brains is expected to be attributable to p39 expression. Consistent with the defective dendritic phenotype observed in *p39*^−/−^ neurons, transcriptomic analysis revealed that p39 knockout mainly affected the cellular processes associated with neuronal morphology. *Rnd1*, *Farp1*, and *Fer* (which encodes Fer, a nonreceptor tyrosine kinase) are differentially regulated genes in *p39*^−/−^ neurons grouped under the “shape changes of neurites” biofunction. Importantly, these genes encode downstream signaling molecules in the Sema3A (class 3A Semaphorin) signaling pathway (Fig. 3c)^29,44,45^, which is well known to mediate both axonal and dendritic development^36^. In addition, the transcript level of another Semaphorin family member, Sema4D, was downregulated in *p39*^−/−^ neurons (Fig. 3c). Of note, the Fyn–Cdk5 axis is a critical downstream signaling pathway of Sema3A that is involved in dendritic orientation in the cerebral cortex^25^. Thus, our results suggest that Cdk5/p39 signaling is critical for dendritic development owing to its regulation of the expression of Semaphorin signaling pathway components. Importantly, WDFY1, one of the most upregulated genes in *p39*^−/−^ neurons, is a downstream effector of NRP-2 (neuropilin 2)^32^, which is the binding receptor for secreted Sema3. Suppression of WDFY1 rescued the defective dendritic phenotype in *p39*^−/−^ neurons, suggesting that the precise control of Cdk5/p39 activity constrains WDFY1 expression and consequently regulates dendritic morphogenesis. Thus, our study suggests that Cdk5/p39 is a critical signaling control of dendrite growth that acts by regulating Semaphorin signaling components.

Multiple cell surface receptors and their corresponding extracellular cues, including Semaphorin/NRP/plexin, reelin/ApoER2/VLDLR, and BDNF/TrkB, play critical roles in the regulation of dendritic morphogenesis^25,36,46–48^. Besides gene transcription, their actions are mediated through the regulation of protein synthesis, protein trafficking, and cytoskeletal reorganization^36^. Concordantly, examination of the altered phosphoproteins in *p39*^−/−^ brains revealed that Cdk5/p39 regulates canonical pathways that are the downstream signaling cascades of Semaphorin/NRP/plexin, reelin/ApoER2/VLDLR, and BDNF/TrkB signaling. In particular, PI3K/Akt- and cAMP-mediated signaling are critical mediators of protein synthesis and cytoskeletal reorganization^49,50^. Moreover, Rho family small GTPases including Rac1 and RhoA play critical roles in axonal and dendritic morphogenesis; they achieve this by regulating actin assembly and organization as well as gene transcription through the activation of downstream effectors^51^. Nonetheless, our results suggest that dendritic morphogenesis involves crosstalk between Cdk5/p39 and multiple signaling transduction pathways.

Phosphoproteomic profiling of *p39*^−/−^ brains revealed the alterations signaling molecules downstream of Cdk5/p39. Accordingly, we identified 3 groups of dysregulated phosphorylated proteins in *p39*^−/−^ brains. The first of such groups contains consensus Cdk5 phosphorylation S/TP sites and were downregulated in *p39*^−/−^ brains. These proteins are potential substrates of Cdk5/p39, and many of them were first reported as possible Cdk5 substrates. The second group of dysregulated phosphorylated proteins consists of phosphorylated peptides that lack Cdk5 phosphorylation sites and were downregulated in *p39*^−/−^ brains. For example, CRMP2, which is critical for mediating microtubule dynamics, undergoes Ser522 priming phosphorylation by Cdk5, which is essential for its sequential phosphorylation by GSK3β and hence the regulation of its activity^52,53^. The third group of dysregulated phosphorylated proteins exhibited increased phosphorylation in *p39*^−/−^ brains. The altered phosphorylation of these proteins might be due to the dysregulation of other kinases in the absence of Cdk5/p39 activity. For example, kinases such as MEK1 (MAP kinase kinase 1) are negatively regulated by Cdk5^54^. Therefore, the dysregulation of Cdk5/p39 might alter the phosphorylation of a network of proteins. Accordingly, unbiased, in-depth transcriptomic and phosphoproteomic profiling are required to characterize the roles of Cdk5/p39 in distinct cellular processes of certain brain functions such as dendritic morphogenesis.

## Conclusions

Our results suggest that Cdk5/p39 acts as a central coordinator to regulate multiple signals from extrinsic cues to cytoskeletal signaling proteins to control the morphology of dendritic trees. Given that the precise control of dendritic arborization is critical for neural network integrity and that the dysregulation of these processes is implicated in various psychiatric and neurodegenerative disorders such as autism and Alzheimer’s disease, our results provide potential insights into the pathogeneses of such disorders.

## Materials and methods

### Animals

*Cdk5*^−/−^ mice were kindly provided by A.B. Kulkarni (National Institutes of Health, Bethesda, MD, USA) and T. Curran (School of Medicine, University of Pennsylvania, Philadelphia, PA, USA), and *p35*^−/−^ and *p39*^−/−^ mice were gifts from L.H. Tsai (Massachusetts Institute of Technology, Cambridge, MA, USA). All animals were bred in the Animal and Plant Care Facility of the Hong Kong University of Science and Technology (HKUST). All experiments were approved by the Animal Ethics Committee of HKUST and conducted in accordance with the Guidelines of the Animal and Plant Core Facility of HKUST.

### Antibodies and constructs

Antibodies against Cdk5 (sc-173, rabbit polyclonal) and p39 (sc-365781, mouse monoclonal) were from Santa Cruz Biotechnologies; antibodies against p35 (#2680, rabbit polyclonal) were from Cell Signaling Technology; antibodies against WDFY1 were from GeneTex (GTX-123058, rabbit polyclonal); and antibodies against α-Tubulin (T9026, mouse monoclonal) and Actin (A5441, mouse monoclonal) were from Sigma-Aldrich. The shRNA sequences and their targets were as follows: rat *Cdk5*, 5′-TGCCACGGGGAGAGACCTG-3′; rat and mouse *p35*, 5′-TATCAACCTCATGAGCTCC-3′; rat, mouse, and human *p39*, 5′-CCTGGTGTTCGTGTACCTG-3′; rat and mouse *Wdfy1* #1, 5′-GCTCCTCAGTGGTTAGAAA-3′ and #2, 5′-GCGATTACTCGGGACAGAT-3′. The shRNAs were cloned into the pSUPER vector and compared to the pSUPER vector control. The knockdown specificity and efficiency of the pSUPER-Cdk5 shRNA construct have already been verified^26^. The knockdown specificities of the *p35*, *p39*, and *Wdfy1* shRNA constructs were confirmed in p35-, p39-, or WDFY1-overexpressing HEK293T cells, respectively (Supplementary Fig. S2a–f), and in rat cortical neurons (Supplementary Fig. S2g–j,S2n,S2o). The RNAi-resistant form of p39 was generated by site-directed mutagenesis, in which the coding sequence at the 238th and 239th positions of human p39 were changed from TTC to TTT and from GTG to GTC, respectively. The expression construct of *Wdfy1* was subcloned from the mouse *Wdfy1* CDS plasmid (MR214818, Origene) to the pcDNA3 vector. The overexpression efficiency of WDFY1 in rat cortical neurons was also confirmed (Supplementary Fig. S2k,S2l). The expression constructs of Cdk5, dominant-negative, kinase-dead Cdk5 (Cdk5-DN), and Cdk5 Y15F mutant have been described elsewhere^26,55^.

### Cell culture and transfection

HEK293T cells were cultured in DMEM (Life Technologies) supplemented with 10% FBS (Life Technologies) and antibiotics. Lipofectamine (Life Technologies) with Plus-based transfection was performed according to the manufacturer’s protocols. Primary hippocampal or cortical neurons prepared from embryonic day 18 (E18) Sprague–Dawley rats or transgenic mice were maintained in Neurobasal medium (Life Technologies) supplemented with 2% B27 (Life Technologies). To examine dendritic morphogenesis, rat hippocampal neurons were transfected with a gene of interest together with a separate construct carrying emGFP at the indicated DIV using the calcium phosphate method as previously described^26^. To evaluate the efficiency of overexpression or knockdown, rat cortical neurons were transfected by nucleofection at 0 DIV using the Rat Neuron Nucleofector Kit (VPG-1003, Lonza) according to the manufacturer’s instructions. The overexpression or knockdown efficiency of neurons at 5 DIV was examined by western blotting.

### Protein extraction and western blotting

The mouse forebrains were homogenized in DPBS containing protease and phosphatase inhibitors, followed by lysis in 2× RIPA lysis buffer. The neurons were directly lysed in 1 × RIPA buffer containing protease and phosphatase inhibitors. The protein lysates were centrifuged at 10,000×*g* at 4 °C for 5 min. The supernatants were saved, and the protein concentration was determined. Equal amounts of protein from each sample were run onto an SDS-PAGE gel, and the separated proteins were transferred to a nitrocellulose membrane. The membrane was blocked with blocking buffer (5% milk in TBS-T) at room temperature for 1 h, followed by incubation with primary antibodies including Cdk5 (1:1000), p35 (1:1000), p39 (1:2000), WDFY1 (1:2000), α-Tubulin (1:10,000), and actin (1:10,000) overnight at 4 °C. The membrane was subsequently incubated with horseradish peroxidase-conjugated secondary antibodies for 1 h. Signal detection was performed using SuperSignal WestPico Chemiluminescent Reagents and exposed to a Fujifilm Super RX X-ray film. The films were developed by a Kodak medical X-ray film processor.

### Golgi staining

Golgi staining was performed using an FD Rapid GolgiStain Kit (PK401, FD NeuroTechnologies) according to the manufacturer’s instructions. In brief, the transgenic mice were deeply anesthetized in an isoflurane-loaded chamber, and their brains were quickly dissected. The brains were then rinsed with water and gently bisected coronally. The brain pieces were incubated with equal volumes of solutions A and B for 10 days and subsequently incubated with solution C (all solutions were provided in the kit) for 3 days. The brain pieces were then slowly frozen in liquid nitrogen-chilled isopentane and sectioned at 160 μm using a CryoStar NX70 Cryostat (Thermo Fisher Scientific). The sections were dried for 2 days before staining with equal volumes of solutions D and E (also provided in the kit) for 10 min. After staining, the sections were subjected to sequential dehydration incubation in 50% ethanol, 70% ethanol, 95% ethanol, 4 times in absolute ethanol, and 3 times in xylene; each incubation step was performed for 4 min. The slides were mounted with Cytoseal 60 mountant (8310, Thermo Fisher Scientific) and dried for 2 days before imaging.

### Image acquisition and quantification

Images of GFP-transfected hippocampal neurons were captured with a 20× dry lens using Leica SP8 confocal microscopy systems. Five to eight serial optical sections (Z-interval: 1 μm) were collected. The maximum projection of the serial images and subsequent analyses were performed using ImageJ (version 1.52a) software. Dendritic morphology was manually traced using the Simple Neurite Tracer^56^ plugin embedded in ImageJ software followed by analyses of neurite number, neurite length, and dendritic tree complexity. Sholl analysis^57^ was performed individually for each neuron from all groups. In brief, the center of the cell soma was set as the center of a series of concentric circles with radii at 10-μm intervals. Then, the intersections between the dendritic tree and the concentric circles were counted. Dendritic morphogenesis from transgenic mouse cortical neurons as indicated by Golgi staining was captured using a Leica DM6000 B compound microscope. The three-dimensional structure of dendritic trees was reconstructed by manual tracing using the filament tool embedded in Imaris (version 8.3.0). Dendrite number and length were also determined using the filament tool.

### RNA extraction and real-time PCR

RNA was extracted using the NucleoSpin RNA column (MACHEREY–NAGEL) according to the manufacturer’s instructions. Single-strand cDNA was first synthesized using a PrimeScript RT Reagent Kit (RR037A, Takara Bio). Quantitative real-time PCR was performed with a Premix Ex Taq Kit (RR390A, Takara Bio) using a 7500 Fast Real-Time PCR System (Applied Biosystems). The mRNA expression was normalized to that of *Hprt*.

### RNA sequencing and data analysis

Cortical neurons from E18 *p39*^−/−^ mouse embryos and their wild-type littermates were cultured until 10 DIV. RNA was extracted using the NucleoSpin RNA column (MACHEREY–NAGEL), and RNA integrity was evaluated by a 2100 Bioanalyzer (Agilent). RNA samples with an RNA integrity number greater than 9 were selected for mRNA enrichment using oligo (dT) beads and fragmentation followed by cDNA library construction by Novogene (Beijing). The transcriptome profile of each sample was generated by Novogene on the Illumina HiSeq X platform. In brief, quality control was performed for the RNA sequencing dataset using FastQC^58^ and RSeQC^59^. Read mapping, counting, and differential gene expression analysis were performed using HISAT2, StringTie^60^, and DESeq2^61^, respectively. The RNA sequencing dataset was submitted to Ingenuity Pathway Analysis (IPA, version 01-13)^62^ to identify the most regulated pathways. The pathways that met the following criteria are shown: activation *z*-score > 1.2 or <  −1.2 and *p* < 0.05.

### Mass spectrometry analysis

The hippocampi of 3 pairs of 1-month-old *p39*^−/−^ mice and their wild-type littermates were processed for iTRAQ labeling by Shanghai Applied Protein Technology. In brief, hippocampal tissues were separately lysed in SDT buffer (4% SDS, 100 mM Tris–HCl, and 1 mM DTT [pH 7.6]) and sonicated. Six samples were labeled using the iTRAQ method and mixed. Phosphorylated peptides were subjected to TiO_2_-based enrichment followed by LC–MS/MS analysis in Thermo Scientific Q Exactive (Shanghai Applied Protein Technology). Unique peptides and protein mapping were performed using Proteome Discovery 1.3 (Thermo Electron) with reference to the UniProt database^63^ (mouse).

Dysregulated phosphoproteins meeting the criterion of *p* < 0.05 were selected for GO analysis based on the PANTHER classification system^37^. Pathway analysis was also performed using IPA in the categories of “biofunction” and “canonical pathways” similar to RNA sequencing analysis. The criteria for the output pathway were as follows: activation z-score > 1.5 or <  − 1.5 and *p* < 0.05.

### Statistical analysis

All data are presented as mean ± SEM. The Mann–Whitney *U-*test or Student’s unpaired *t*-test was performed where appropriate to evaluate the significance of differences between 2 experimental conditions. Kruskal–Wallis one-way ANOVA followed by Dunn’s test was performed where appropriate to evaluate the significance of differences of 3 or more experimental conditions. All experiments were performed independently at least 3 times or on 3 animals per group.

## Supplementary information

Supplementary Information 1.

Supplementary Information 2.

Supplementary Information 3.
