# Supplementary material for: p39-associated Cdk5 activity regulates dendritic morphogenesis
Source: Sci Rep. 2020 Oct 30;10:18746. doi: 10.1038/s41598-020-75264-6 (PMC7603351; doi:10.1038/s41598-020-75264-6)

SUPPLEMENTARY MATERIAL

p39-associated Cdk5 activity regulates dendritic morphogenesis

Li Ouyang[1,2,†], Yu Chen[1,2,3,4,†], Ye Wang[1,2], Yuewen Chen[1,2,3,4], Amy K.Y. Fu[1,2,4], Wing-Yu Fu[1,2], Nancy Y. Ip[1,2,4,*]

[1] Division of Life Science, State Key Laboratory of Molecular Neuroscience and Molecular Neuroscience Center, The Hong Kong University of Science and Technology, Clear Water Bay, Hong Kong, China

[2] Hong Kong Center for Neurodegenerative Diseases, Hong Kong Science Park, Hong Kong, China

[3] The Brain Cognition and Brain Disease Institute, Shenzhen Institute of Advanced Technology, Chinese Academy of Sciences; Shenzhen-Hong Kong Institute of Brain Science-Shenzhen Fundamental Research Institutions, Shenzhen, Guangdong 518055, China

[4] Guangdong Provincial Key Laboratory of Brain Science, Disease and Drug Development, HKUST Shenzhen Research Institute, Shenzhen–Hong Kong Institute of Brain Science, 518057, Shenzhen, Guangdong, China

[†] These authors contributed equally to the manuscript

[*] Correspondence and requests for materials should be addressed to N.Y.I. (email: boip@ust.hk)

Supplementary Figure S1

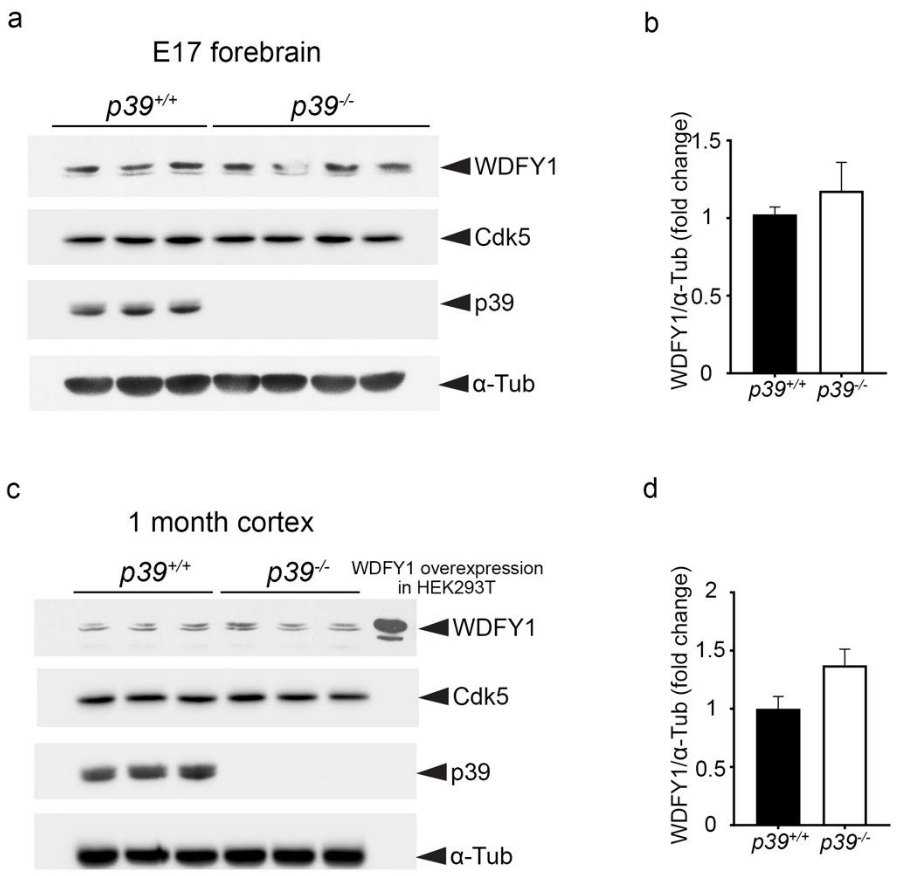

Supplementary Figure S1. Protein expression of the Cdk5–p39–WDFY1 axis in p39-knockout mouse brains. Brain lysates were collected from embryonic day 17 (E17) forebrains (a, b) and 1-month-old cortices (c, d) of p39-knockout mice followed by western blotting against WDFY1, Cdk5, p39, and α-tubulin (α-Tub). The expression of WDFY1 is normalized to that of α-tubulin.

Supplementary Figure S2

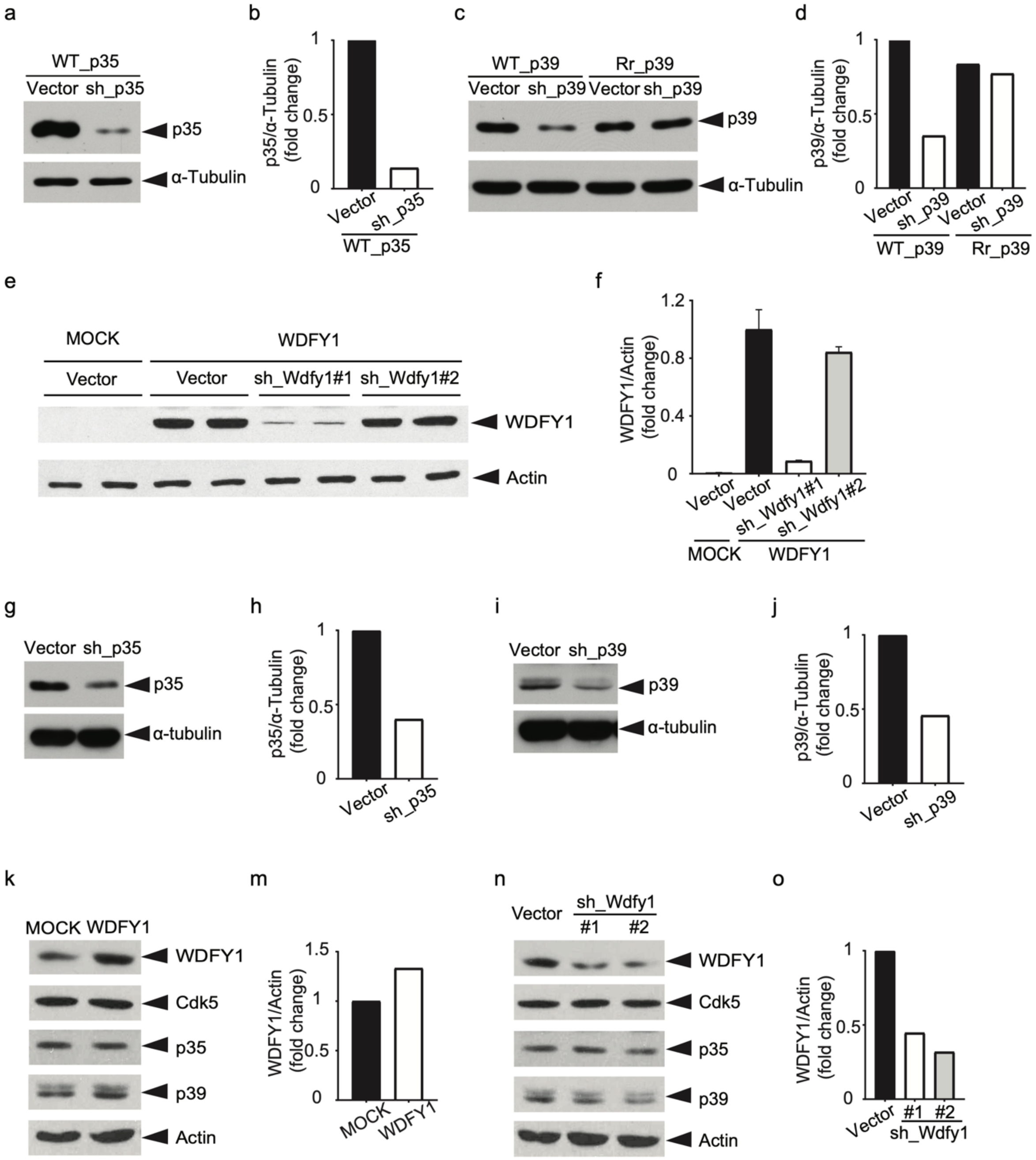

Supplementary Figure S2. Knockdown and overexpression efficiencies of shRNA and overexpression constructs. Knockdown efficiency was performed in HEK293T cells by co-transfection with shRNA and overexpression constructs. (a, b) p35 expression in p35-overexpressing HEK293T cells was reduced by the sh_p35 construct. (c, d) p39 expression in p39-overexpressing HEK293T cells was knocked down by the sh_p39 construct. The protein level of the RNAi-resistant form of p39 (Rr_p39) was unaffected by sh_p39. (e, f) WDFY1 expression is increased by overexpression of WDFY1 construct compared to that of pcDNA vector as indicated as “MOCK”. WDFY1 expression in WDFY1-overexpressing HEK293T cells was significantly reduced by the sh_Wdfy1#1 construct. (g–n) The knockdown efficiencies of shRNAs against *p35* (g, h), *p39* (i, j), and *Wdfy1* (m, n) constructs were examined in rat cortical neurons by nucleofection at 0 days in vitro, and the efficiencies were examined at 5 days in vitro. (k, l) Overexpression efficiency of the *Wdyf1* construct in rat cortical neurons. The data were normalized to the expression of α-tubulin or actin as indicated.

Supplementary information

Raw images for western blotting

Figure 3g and 3h

3g

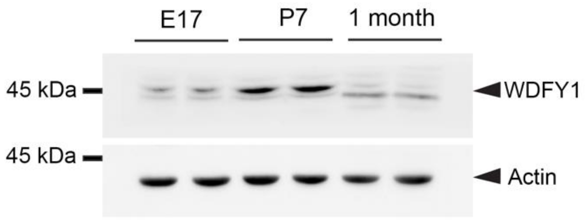

3h

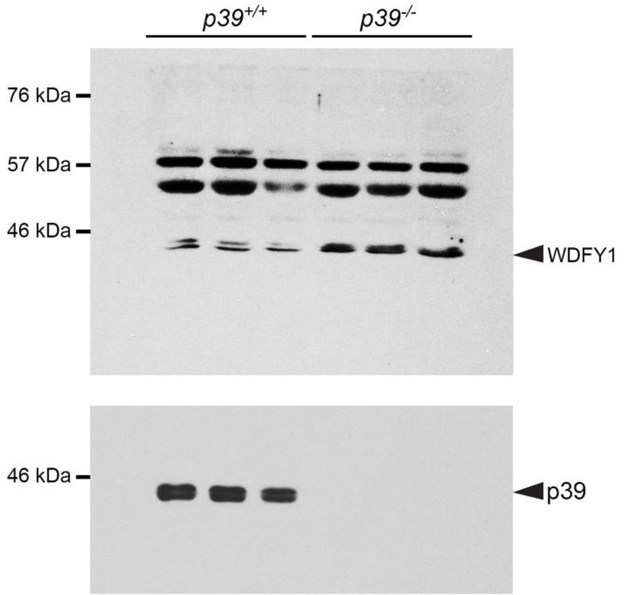

Figure 4a

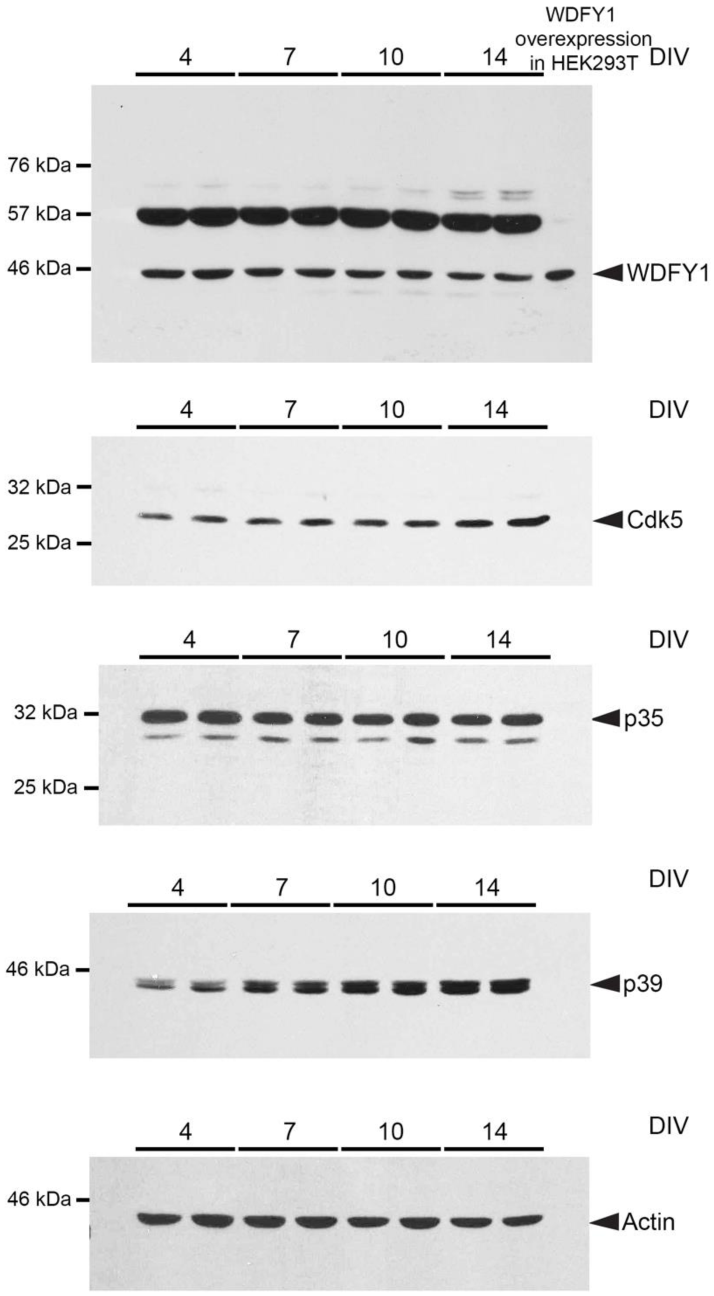
WDFY1 overexpression in HEK293T
4
7
10
14
DIV
76 kDa
57 kDa
46 kDa
WDFY1
32 kDa
25 kDa
Cdk5
p35
p39
Actin

Figure S1a and S1c

S1a

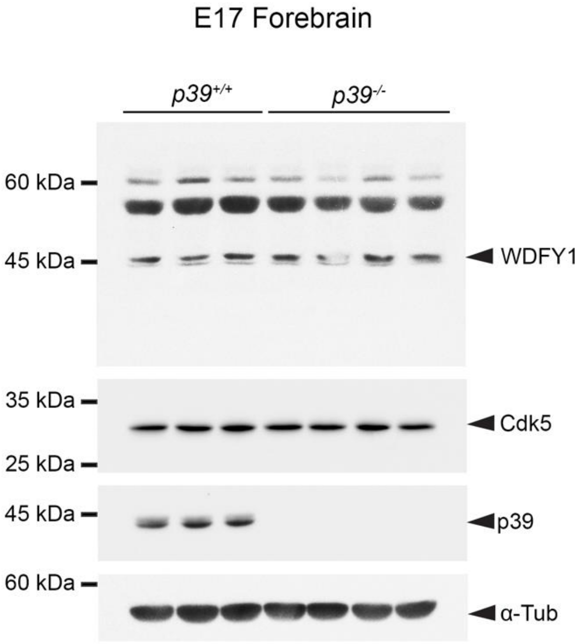

S1c

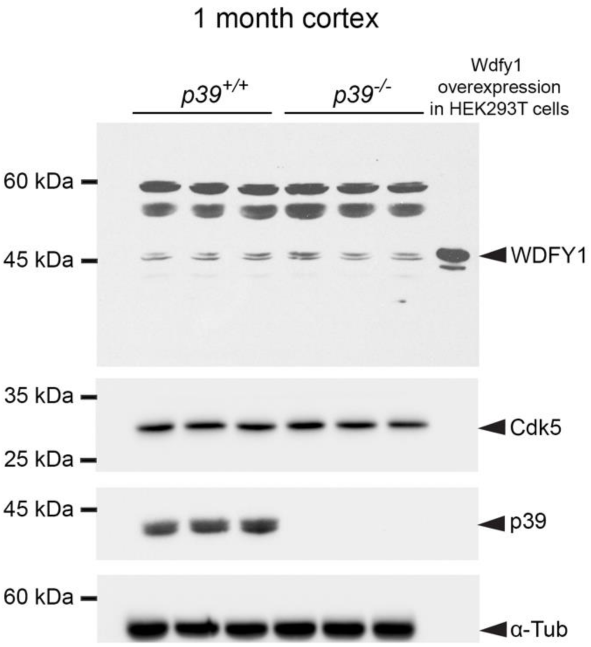

Figure S2a

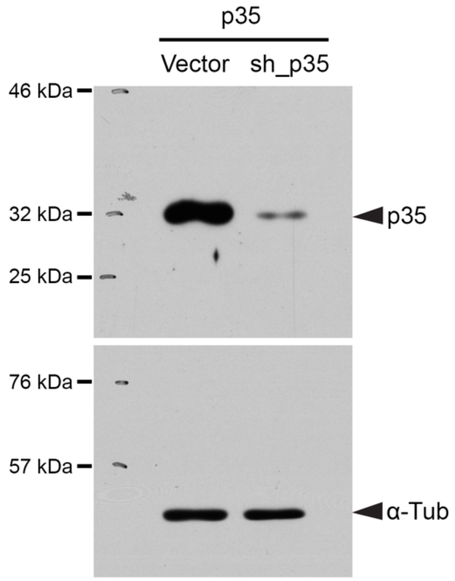

Figure S2c

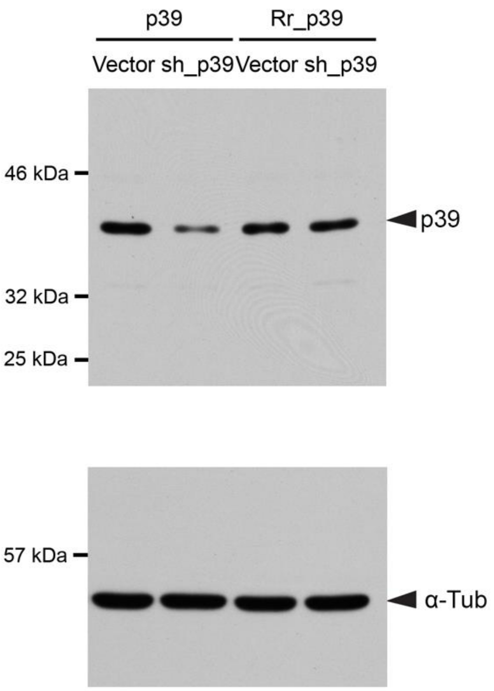
p39
Rr_p39
Vector sh_p39 Vector sh_p39
46 kDa
32 kDa
25 kDa
p39
57 kDa
α-Tub

Figure S2e

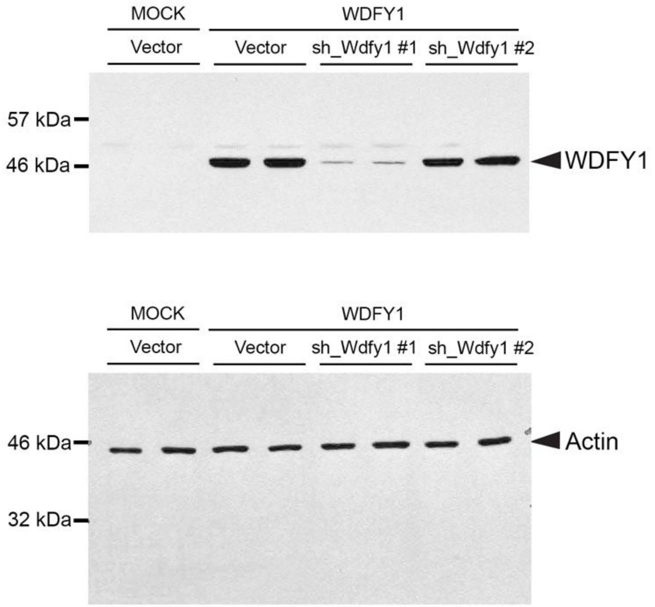
MOCK
WDFY1
Vector
Vector
sh_Wdfy1 #1
sh_Wdfy1 #2
57 kDa
46 kDa
WDFY1
MOCK
WDFY1
Vector
Vector
sh_Wdfy1 #1
sh_Wdfy1 #2
46 kDa
32 kDa
Actin

Figure S2g

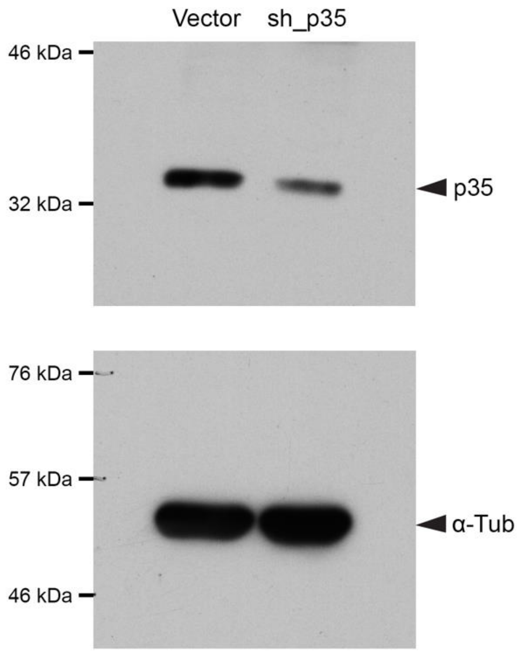

Figure S2i

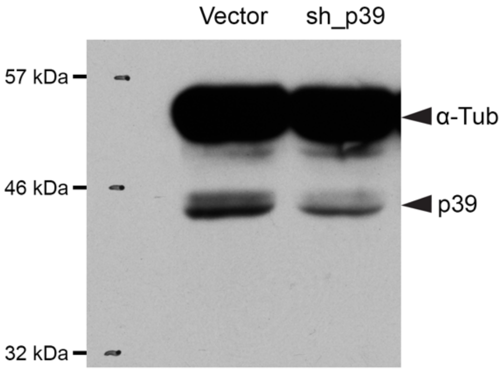

Figure S2k and S2m

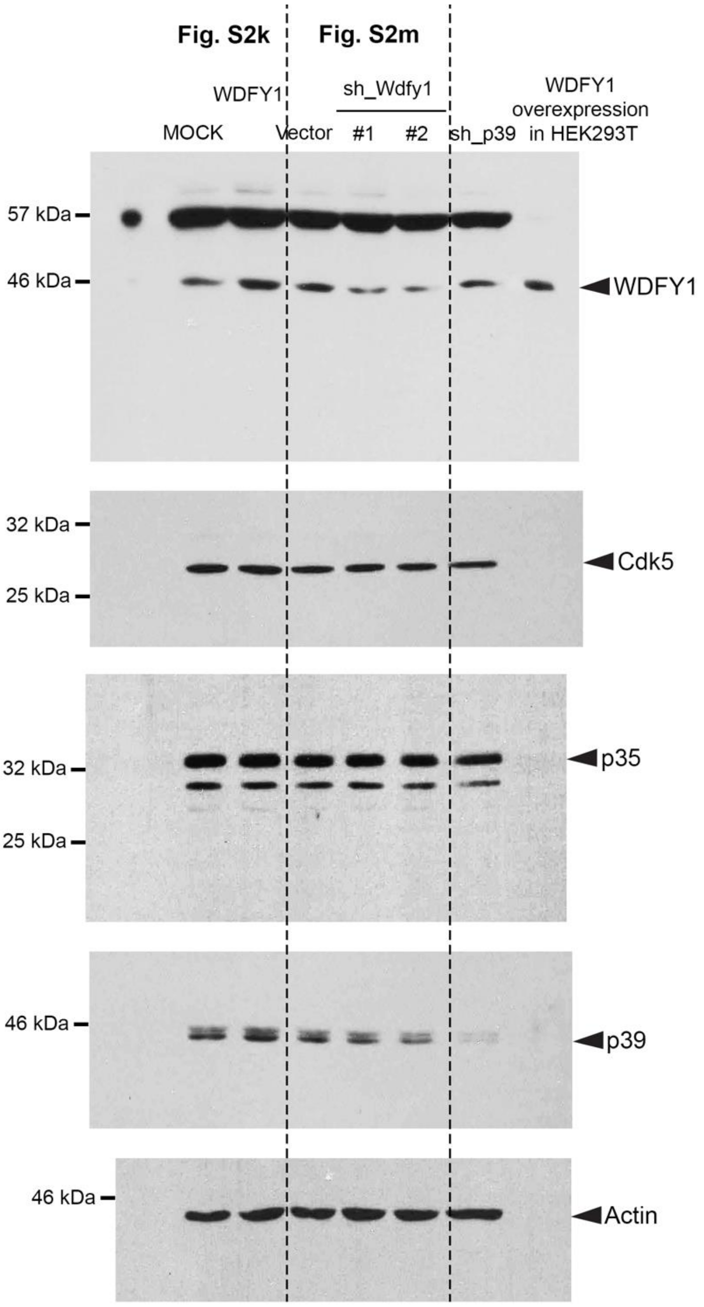

Supplement: Supplementary file 1 — Supplementary Information 1. [file 41598_2020_75264_MOESM1_ESM.pdf]
